# The Influence of Preoperative Nutritional and Systemic Inflammatory Status on Perioperative Outcomes following Da Vinci Robot-Assisted Thoracic Lung Cancer Surgery

**DOI:** 10.3390/jcm12020554

**Published:** 2023-01-10

**Authors:** Camilo Moreno, Anna Ureña, Ivan Macia, Francisco Rivas, Carlos Déniz, Anna Muñoz, Ines Serratosa, Violeta Poltorak, Miguel Moya-Guerola, Cristina Masuet-Aumatell, Ignacio Escobar, Ricard Ramos

**Affiliations:** 1 Department of Thoracic Surgery, Hospital Universitari de Bellvitge, Bellvitge Biomedical Research Institute (IDIBELL), L’Hospitalet de Llobregat, 08907 Barcelona, Spain; 2 Unit of Human Anatomy, Department of Pathology and Experimental Therapeutics, Medical School, University of Barcelona, L’Hospitalet de Llobregat, 08907 Barcelona, Spain; 3 Department of Preventive Medicine, Hospital Universitari de Bellvitge, Bellvitge Biomedical Research Institute (IDIBELL), L’Hospitalet de Llobregat, 08907 Barcelona, Spain

**Keywords:** robotic surgery, early-stage lung cancer, body mass index, nutritional status, systemic inflammatory status

## Abstract

Background: Nutrition is an important factor in the outcome of any disease process. We evaluated the relationship of nutritional status and inflammatory status of non-small cell lung cancer (NSCLC) patients undergoing robotic-assisted thoracic surgery (RATS) with postoperative complications. Methods: This prospective cohort study included 107 NSCLC patients undergoing surgical treatment, between 2019 and 2021. Nutritional status and inflammatory status were assessed before pulmonary resection using anthropometric assessment, blood tests, and body mass index (BMI). Results: The BMI was 27.5 ± 4.4. Based on BMI, 29% (*n* = 31) were classified as normal weight, 43% (*n* = 46) as overweight, and 28% (*n* = 30) as obese. The mean neutrophil/lymphocyte ratio (NLR) was 2.16 ± 0.85, the platelet/lymphocyte ratio (PLR) was 121.59 ± 44.21, and the lymphocyte/monocyte ratio (LMR) was 3.52 ± 1.17. There was no increase in the number of intraoperative complications or bleeding (*p* = 0.696), postoperative complications (*p* = 0.569), mean hospital stay (*p* = 0.258) or duration of chest drain (*p* = 0.369). Higher inflammatory status, with an NLR > 1.84, was associated with more overall postoperative complications (*p* = 0.028), only in univariate analysis, but this significance was not maintained on multivariate analysis. Conclusions: BMI was not a predictor of increased postoperative risk in this cohort; therefore, weight should not deter surgeons from using RATS for pulmonary resection.

## 1. Introduction

According to the World Health Organization (WHO), obesity is defined as abnormal or excessive accumulation of body fat that may impair health [1]. It has become a leading problem in health care [2] and affects 10–30% of adults in European countries [3]. Twenty-six percent of patients who undergo lung resection are obese [4].

The association between overweight and obesity and other chronic pathologies such as hypertension, dyslipidemia, and diabetes—which can increase the postoperative risk after any type of cancer surgery—is well known [5]. In addition, Thomas et al. [6] found that malnutrition increased the risk of some complications immediately after lung cancer surgery, concluding that preoperative nutritional status should be taken into account and improved as much as possible prior to surgery. Our team studied the close association between preoperative nutritional status and postoperative outcomes in lung resection surgery and found that patients with low weight had more postoperative complications [7]. The “obesity paradox” refers to a better prognosis in obese patients compared to normal or underweight patients [8,9].

Another prognostic factor that has been associated with survival and complications is inflammatory status. Multiple parameters can be used to determine the inflammatory status, but blood markers that are often used in preoperative assessment, due to being simple to obtain, are absolute values of neutrophils, lymphocytes, monocytes, and platelets, and the ratios of neutrophils to lymphocytes (NLR), platelets to lymphocytes (PLR), and lymphocytes to monocytes (LMR) [10,11]. Our team previously concluded that preoperative LMR was an independent prognostic factor of overall survival and recurrence-free survival in patients with early-stage lung cancer that was surgically resected through an open approach [12].

The aim of this prospective study was to assess the impact of preoperative nutritional and systemic inflammatory status on postoperative outcomes in patients undergoing robotic anatomical lung resections. We used BMI, NLR, PLR, and LMR to stratify patients according to their nutritional and systemic inflammatory status and examined the predictive value of these factors on postoperative complications.

## 2. Materials and Methods

### 2.1. Study Population

This prospective study included a cohort of 138 patients who were treated with robotic radical lung resection between February 2019 and December 2021. Patients with a history of systemic inflammatory disease, concomitant active infection, neoadjuvant treatment, preoperative stage ≥ T3, preoperative stage ≥ N1, patients lost to follow-up, uniportal robot-assisted thoracic surgery (RATS), or those for whom preoperative blood tests were not available were excluded. Finally, we included 107 patients with early clinical stage non-small cell lung cancer (NSCLC) who underwent anatomic pulmonary resection with systematic lymph node dissection. In patients with functional contraindication for lobectomy (preoperative %DLCO < 50% or VO2 max between 10 and 15 mL/kg/min), a sublobar resection was performed. All patients gave signed informed consent, and this study was approved by the Institutional Review Board (PR30416). All patients underwent full robotic thoracic surgery (da Vinci Xi robot system1—Intuitive Surgical Sarl, Aubonne, Switzerland) combined with standard hilar and mediastinal lymphadenectomy.

### 2.2. Assessment of Nutritional and Systemic Inflammatory Status

Nutritional status was assessed prospectively before surgery based on demographic data (age and sex) and anthropometric evaluation (weight and height). Patients were classified into the following four groups according to their BMI: underweight (BMI < 18.5 kg/m^2^), normal weight (BMI 18.5–24.9 kg/m^2^), overweight (BMI 25–29.9 kg/m^2^), and obese (BMI ≥ 30 kg/m^2^). Obese patients were not subdivided according to degree of obesity.

Systemic inflammatory status was assessed prospectively before surgery based on the neutrophil-to-lymphocyte ratio (NLR), platelet-to-lymphocyte ratio (PLR), and lymphocyte-to-monocyte ratio (LMR), calculated from the absolute neutrophil, platelet, monocyte, and lymphocyte counts from routine preoperative testing performed 1–2 weeks before surgery at the same hospital.

### 2.3. Study Variables

The clinical variables and postoperative clinical course were stratified according to BMI and systemic inflammatory parameters. The study variables included the following: age, sex, comorbidities (smoking, diabetes mellitus, ischemic heart disease, chronic obstructive pulmonary disease, dyslipidemia, and hypertension), type of surgery performed, pathological stage, tumor histology, postoperative complications in hospital, 30-day postoperative mortality, and hospital length of stay (LOS) measured from the day of surgery to discharge.

Postoperative complications included the following: atrial fibrillation, pneumonia, respiratory failure, hemothorax, and persistent air leak. A diagnosis of pneumonia was based on the presence of clinical and laboratory signs of lung infection, together with radiographic findings consistent with pneumonia. Respiratory failure was characterized for 2 of these 3 criteria: partial pressure of oxygen (PaO2) less than 60 mmHg (or room air oxygen saturation less than 91%), and partial pressure of carbon dioxide (PaCO2) greater than 50 mmHg with pH less than 7.35 and signs/symptoms of respiratory distress. Wound infection was defined as the presence of positive bacterial culture from the surgical wound with clinical signs of infection. Persistent air leak was defined as the presence of leak on or after postoperative day 5. A digital chest drainage system (Medela-Thopaz; Medela AG, Switzerland) was used in all patients. The drainage tube was removed 24 h after air leaks were no longer detectable and the drainage volume was <100 mL. The chest drain duration was calculated from the day of insertion to the day of removal.

All of the patients were admitted to the hospital on the day the surgical intervention was scheduled. The management of postoperative pain consisted of serratus and intercostal plane block with non-steroidal anti-inflammatories and rescue opioids. Antithrombotic prophylaxis, in the form of subcutaneous injection of low-molecular-weight heparin, was initiated 24 h after surgery and continued until discharge.

### 2.4. Surgical Technique

Under general anesthesia and with double-lumen endotracheal intubation for single-lung ventilation, three robotic operating ports of 8 mm and 1 port of 12 mm were placed in the 7th or 8th intercostal space. The lung was then deflated, and CO_2_ insufflation was set at 5–10 mmHg through the non-camera port with careful hemodynamic monitoring. A 30-degree endoscope was placed, and the lung, pleural cavity, and mediastinum were explored; subsequently, fenestrated bipolar (EndoWrist^®^ Bipolar, Intuitive, Sunnyvale, CA, USA) and Maryland bipolar forceps (EndoWrist^®^ Bipolar) were used for dissection and Cadiere forceps were used to separate the lung. Anatomic pulmonary resection was performed following the usual oncological technique, and a 28-F chest tube was left in place after expansion of the lung under direct visualization.

### 2.5. Statistical Analysis

The distribution of the variables was evaluated using the Kolmogorov–Smirnov test. For quantitative variables following a non-normal distribution, the results were expressed as medians and interquartile ranges (IQR) and compared using the Mann–Whitney U test. For quantitative variables following a normal distribution, the results were expressed as means and standard deviations and compared using the Student t-test. Between-group comparisons were performed using the Chi-squared test for qualitative variables. Logistic regression was performed to analyze the relationship between variables of interest. Univariate analyses were also conducted to determine the risk of complications. Subsequently, independent variables (NLR, BMI) were included in a multivariate logistic regression model to adjust for potential confounders (age, sex, pathological stage, and histology) and estimate the likelihood of complications. To perform logistic regression analysis, those variables with a *p*-value equal to or less than 0.25 or of clinical interest were, therefore, considered for inclusion. A stepwise backward selection procedure was used, fixing a *p*-value of ≤0.40 as the criterion for removing variables from the final multivariate regression models. The results were reported as crude and adjusted odds ratios (ORs) and 95% confidence intervals (CIs). A receiver operating characteristics (ROC) analysis was performed to calculate the NLR, PLR, and LMR values that would have the greatest sensitivity and specificity. All of the statistical tests were two-tailed, and a *p*-value ≤0.01 was considered statistically significant. Data were analyzed using the Statistical Software Package SPSS 28.0 (SPSS, Inc., Chicago, IL, USA).

## 3. Results

The study population included 107 consecutive patients with early-stage NSCLC whose tumors were surgically resected in a single center. Most of the patients were men (65, 60.7%), and the median age was 70 (IQR 12) years. The mean BMI was 27.5 ± 4.4, and the mean NLR, PLR, and LMR were 2.16 ± 0.85, 121.59 ± 44.21, and 3.52 ± 1.17, respectively. The overall mean operating room (OR) time for all patients was 150 min, with a median estimated blood loss of 50 (IQR 130) mL, a complication rate of 42.05%, a conversion rate of 0.9%, an air leak rate of 31.8%, and a length of stay of 5 days. Table 1 shows the clinicopathological characteristics, surgical data, nutritional parameters, and systemic inflammatory parameters of the study population.

Higher BMI values were significantly correlated with vascular disease. No associations were found between BMI and disease stage, type of lung resection, or pathological features. We found no significant differences between the different BMI groups in terms of postoperative complications, chest-drainage duration, or LOS (Table 2).

Following ROC analysis, the cutoff values with the greatest sensitivity and specificity were 1.84 for NLR, 3.27 for LMR, and 115.2 for PLR. The AUC for NLR was 0.601; 95% CI, 0.409–0.791, with a sensitivity of 66.7% and a specificity of 53.8%. However, the ROC analysis of PLR and LMR found that these two variables were not robust enough to predict complications in robotic surgery because of a non-significant AUC (PLR AUC 0.514; 95% CI, 0.399–0.629; LMR AUC 0.461; 95% CI, 0.350–0.572) and low internal validity scores. 

On univariate analysis, NLR ≥ 1.84 was significantly associated with more overall postoperative complications (*p* = 0.028) (Table 3), but this significance was not maintained on multivariate analysis (HR, 2.283; 95% CI, 0.831–6.271; *p* = 0.109) after adjusting for age, sex, pathological stage, and histology (Table 4).

## 4. Discussion

The main objective of this study was to determine whether preoperative nutritional and systemic inflammatory status—assessed by BMI and NLR—could predict the likelihood of postoperative complications in patients undergoing robotic lung resection for cancer. The main findings of this study were that the preoperative BMI was not associated with postoperative complications and that preoperative NLR, on univariate analysis, was associated with postoperative complications, although this was not maintained in the multivariate analysis. However, we cannot discard that preoperative BMI and NLR might affect the incidence of postoperative complications.

According to the World Health Organization, obesity (BMI > 30 kg/m^2^) is a global health problem, with a high prevalence that tripled between 1975 and 2016. Classically, it was a problem of rich countries, although it is increasingly common in countries with middle or low income. The association between overweight or obesity and chronic diseases such as diabetes, ischemic heart disease, and others is clear, and such diseases in turn increase the risk of postoperative complications [1]. For several years now, the presence of obesity (BMI > 25 kg/m^2^) in the general population has also been observed in patients with cancer who are candidates for surgery [13], as supported by our current results as well as those described in a previous study [7]. Although the Mediterranean diet is common in Southern European countries and has some clear benefits compared with diets from Anglo-Saxon countries, we still observe a high number of patients who are overweight or obese.

The effect of nutritional status, be it malnutrition or obesity, on postoperative outcomes in patients undergoing lung resection is known to be relevant and has been discussed and studied, with some contradictory results [14,15,16,17]. Extreme values [18] of either malnutrition or obesity are associated with greater postoperative complications, while overweight and mild obesity have a protective effect, known as “the obesity paradox” [8,9]. Li et al. [19] observed that obesity was a protective factor in terms of immediate postoperative outcomes as well as survival in patients undergoing open surgery, while our team observed more respiratory complications in patients with low weight and improved survival in a series of patients who were overweight and underwent open surgery [7].

Another aspect related to nutritional status is the patient’s systemic inflammatory status. Blood-marker values can behave differently in Asian, European, or American populations, so the results will have to be validated in each study population, using receiver operating characteristic (ROC) curve analysis. Our team described, in a series of patients undergoing open surgery, that a lymphocyte/monocyte ratio ≥ 2.5 was an independent favorable prognostic factor in terms of disease-free survival and overall survival [12], although we did not study this as a marker of immediate postoperative complications. In the present study, we observed that an NLR > 1.84 was associated with immediate postoperative complications, with similar results to those described by other authors [11,20], although this was not maintained in the multivariate analysis, possibly due to the number of patients of the study. However, we cannot discard that preoperative NLR might affect the incidence of postoperative complications.

Cancer surgery has evolved from open surgery, with a high number of postoperative complications in patients with overweight or obesity, to minimally invasive surgery, which has already been shown to have similar oncological outcomes in terms of survival [21,22] and fewer postoperative complications [23,24]. Gómez-Hernández et al. [25] observed more postoperative respiratory complications in overweight and obese patients with lung cancer undergoing video-assisted thoracic surgery (VATS) and RATS; this differs from our results, in patients who had exclusively RATS. Montané et al. [26] reported results similar to ours in a group of patients with overweight or obesity who underwent RATS.

Compared with open surgery, RATS provides the surgeon with better visualization and a range of surgical movements as well as better accessibility, and clear advantages in terms of early postoperative outcomes, especially in obese patients [27], although there are no studies that compare against VATS.

## 5. Limitation of the Study

The main limitations of our study are the number of patients who had robotic surgery; the outcomes were not compared against VATS and the non-assessment of other systemic inflammatory markers.

## 6. Conclusions

The main limitations of our study are the number of patients who had robotic surgery and that the outcomes were not compared against VATS. Our aim was to assess the outcomes after robotic surgery in a group of patients that is increasingly common in thoracic surgery clinics; we observed that weight should not discourage the surgeon from a robotic approach and confirmed, in this series of patients, that the high systemic inflammatory state does not lead to more postoperative complications overall following robotic surgery.

## Figures and Tables

**Table 1:** Clinical, pathological, surgical, nutritional, and systemic inflammatory parameters of the study population.

Baseline Characteristics (N = 107)		N (%) or Mean ± SD or Median (IQR)
Males		65(60.7%)
Age (years)		70(12)
High blood pressure		52(48.6%)
Diabetes		17(15.9%)
Dyslipidemia		43(40.2%)
COPD		18(16.8%)
Ischemic cardiopathology		11(10.3%)
Smokers		77 (72%)
BMI (kg/m^2^)	Normal weight (18.5–24.9)	31 (29%)
	Overweight (25–29.9)	46 (43%)
	Obesity (>30)	30 (28%)
PLR		121.59 ± 44.21
NLR		2.16 ± 0.85
LMR		3.52 ± 1.17
Surgical procedure: lobectomy		97 (90.7%)
Tumor size (mm)		23.53 ± 11.00
Number of lymph nodes		15.29 ± 7.08
Histological type	Adenocarcinoma	60 (56.1%)
	Squamous cell carcinoma	21 (19.6%)
	Others	26 (24.3%)
Surgical duration (minutes)		150 (51)
Bleeding (ml)		50 (130)
Hospitalization days		5.0 (3.0)
Chest drainage days		4.0 (4.0)
Pathologic Stage TNM 8th	0	4 (3.7%)
	I	68 (63.6%)
	II	15 (14%)
	III	5 (4.7%)
	IV	1 (0.9%)

COPD: Chronic obstructive pulmonary disease; BMI: body mass index; PLR: platelet-to-lymphocyte ratio; NLR: neutrophil-to-lymphocyte ratio; and LMR: lymphocyte-to-monocyte ratio.

**Table 2:** Clinical, pathological, and surgical parameters, and postoperative complications, according to BMI.

		BMI (kg/m^2^) (*n* = 107)			
N (%) or Mean ± SD or Median (IQR)		Normal Weight *n* = 31 (18.5–24.9)	Overweight *n* = 46 (25–29.9)	Obesity *n* = 30 (>30)	*p*-Value
Males		22 (71%)	29 (63%)	14 (46.7%)	0.138
Age (years)		65.39 ± 9.59	65.69 ± 9.69	68.73 ± 7.97	0.303
High blood pressure		15 (48.4%)	24 (52.2%)	13 (43.3%)	0.752
Diabetes		8 (25.8%)	3 (6.5%)	6 (20%)	0.058
Dyslipidemia		12 (38.7%)	16 (34.8%)	15 (50%)	0.409
COPD		6 (19.4%)	7 (15.2%)	5 (16.7%)	0.893
Ischemic cardiopathology		3 (9.7%)	6 (13%)	2 (6.7%)	0.664
Smokers		25 (80.6%)	34 (73.9%)	18 (60%)	0.185
PLR		125.19 ± 51.07	116.81 ± 40.68	125.22 ± 44.83	0.449
NLR		2.11 ± 0.96	2.03 ± 0.74	2.37 ± 0.91	0.449
LMR		3.54 ± 1.49	3.64 ± 1.10	3.34 ± 1.00	0.484
Surgical procedure: lobectomy		29 (93.5%)	42 (91.3%)	26 (86.7%)	0.64
Tumor size (mm)		19.13 ± 7.09	25.52 ± 11.00	24.44 ± 13.04	0.185
Number of lymph nodes		15 ± 8.08	15.68 ± 5.88	15.00 ± 8.07	0.603
Histological type	Adenocarcinoma	20 (64.5%)	25 (54.3%)	15 (50%)	0.33
	Squamous cell carcinoma	6 (19.4%)	8 (17.4%)	7 (23.3%)	
	Others	5 (16.1%)	13 (28.3%)	8 (26.7%)	
Surgical duration (minutes)		157.53 ± 36.89	150.88 ± 39.69	156.78 ± 33.63	0.224
Bleeding (mL)		2.99 (2.01)	3.91 (1.96)	4.00 (2.09)	0.696
Hospitalization days		6.33 ± 4.73	5.64 ± 3.23	6.00 ± 5.33	0.369
Chest drainage days		5.00 (4.00)	5.00 (4.00)	5.00 (2.00)	0.258
Persistent air leak		10 (32.3%)	16 (34.8%)	8 (26.7%)	0.757
Pleuropulmonary infection		1 (3.2%)	3 (6.5%)	0 (0%)	0.337
Postoperative atrial fibrillation		0 (0%)	1 (2.2%)	1 (3.3%)	0.617
Hemothorax		1 (3.2%)	2 (4.3%)	0 (0%)	0.525
Reconversion		1 (3.2%)	0 (0%)	0 (0%)	0.295
Reoperation		0 (0%)	2 (4.3%)	0 (0%)	0.259
Readmission		0 (0%)	1 (2.2%)	0 (0%)	0.497
Death		1 (3.2%)	1 (2.2%)	0 (0%)	0.636
Postoperative complications		12 (38.7%)	22 (47.8%)	11 (36.7%)	0.569
Pathologic Stage TNM 8th	0	1 (3.7%)	2 (5.1%)	1 (3.7%)	0.067
	I	18 (66.7%)	30 (76.9%)	20 (74.1%)	
	II	7 (25.9%)	3 (7.7%)	5 (18.5%)	
	III	1 (3.7%)	3 (7.7%)	1 (3.7%)	
	IV	0 (0%)	1 (2.6%)	0 (0%)	

COPD: chronic obstructive pulmonary disease; BMI: body mass index; PLR: platelet-to-lymphocyte ratio; NLR: neutrophil-to-lymphocyte ratio; and LMR: lymphocyte-to-monocyte ratio.

**Table 3:** Postoperative complications according to clinical, pathological, and systemic inflammatory parameters and surgical analysis.

N (%) or Mean ± SD or Median (Range)		Complications		
(N = 107)		No	Yes	*p*-Value
Males		36 (58.1%)	29 (64.4%)	0.505
Age (years)		66.53 ± 8.97	67.77 ± 7.99	0.139
High blood pressure		30 (48.4%)	22 (48.9%)	0.959
Diabetes		9 (14.5%)	8 (17.8%)	0.649
Dyslipidemia		28 (45.2%)	15 (33.3%)	0.218
COPD		6 (9.7%)	12 (26.7%)	0.2
Ischemic cardiopathology		5 (8.1%)	6 (13.3%)	0.376
Smokers		42 (67.7%)	35 (77.8%)	0.254
BMI (kg/m^2^)	Normal weight (18.5–24.9)	19 (30.6%)	12(26.7%)	0.569
	Overweight (25–29.9)	24 (38.7%)	22(48.9%)	
	Obesity (>39)	19 (30.6%)	11(24.4%)	
NLR >1.84 (yes)		28 (45.2%)	30 (66.67%)	0.028
Surgical procedure: lobectomy		55 (88.7%)	42 (93.3%)	0.417
Tumor size (mm)		21.69 ± 10.41	26.55 ± 11.52	0.257
Number of lymph nodes		15.11 ±7.98	15.59 ± 5.45	0.9
Histological type	Adenocarcinoma	36 (58.1%)	24 (53.3%)	0.383
	Squamous cell carcinoma	10 (16.1%)	11 (24.4%)	
	Others	16 (25.8%)	10 (22.3%)	
Surgical duration (minutes)		150.00 (58.00)	152.50 (50.00)	0.057
Bleeding (mL)		20.00 (46.00)	85.00 (173.00)	0.612
Hospitalization days		4.00 (1.00)	9.50 (4.00)	<0.001
Chest drainage days		4.00 (1.00)	9.00 (5.00)	<0.001
Pathologic Stage TNM 8th	0	3 (5.8)	1 (2.4)	0.65
	I	39 (75)	29 (70.7)	
	II	7 (13.5)	8 (19.5)	
	II	3 (5.8)	2 (4.9)	
	IV	0 (0)	1 (2.4)	

COPD: Chronic obstructive pulmonary disease; BMI: body mass index; PLR: platelet-to-lymphocyte ratio; NLR: neutrophil-to-lymphocyte ratio; and LMR: lymphocyte-to-monocyte ratio.

**Table 4:** Multivariate analysis adjusted by age, sex, pathological stage, and histology.

	Complications	No Complications	*p*	ORa	95% CI	P ^a^
NLR > 1.84	30 (66.67%)	28 (45.2%)	0.028	2.283	(0.831–6.271)	0.109
BMI (kg/m^2^)						
Normal	19 (30.6%)	12 (26.7%)	0.569			0.529
Overweight	24 (38.7%)	22 (48.9%)		1.587	(0.500–5.043)	0.433
Obesity	19 (30.6%)	11 (24.4%)		0.838	(0.207–3.391)	0.804

BMI: Body mass index; NLR: neutrophil-to-lymphocyte ratio; ORa: adjusted odds ratio; And CI: confidence interval. ^a^ Adjusted by age, sex, pathological stage, and histology.

## Data Availability

The pseudonymized data presented in this study are available on request from the corresponding author. The data are not publicly available due to legal restrictions in Spain.
